# 3D analysis of child facial dimensions for design of medical devices in low-middle income countries (LMIC)

**DOI:** 10.1371/journal.pone.0216548

**Published:** 2019-05-23

**Authors:** Israel Amirav, Claude Kasereka Masumbuko, Michael T. Hawkes, Ian Solomon, Yossi Aldar, Gil Margalit, Alon Zvirin, Yaron Honen, Eugenie Sahika Sivasivugha, Ron Kimmel

**Affiliations:** 1 Department of Pediatrics, University of Alberta, Edmonton, Canada; 2 Association for Health Innovation in Africa, Butembo, Democratic Republic of the Congo; 3 Université Catholique du Graben, Butembo, Democratic Republic of the Congo; 4 RespiDx Ltd., Tel Aviv, Israel; 5 Tel-Aviv University, Tel Aviv, Israel; 6 Department of Computer Sciences, Technion University, Haifa, Israel; Indraprastha Institute of Information Technology, INDIA

## Abstract

**Background:**

Facial anthropometric data are scarce in African children. However, such data may be useful for the design of medical devices for high disease burden settings. The aim of this study was to obtain 3D facial anthropometric data of Congolese children aged 0–5 years.

**Methods & findings:**

The faces of 287 Congolese children were successfully scanned using a portable structured-light based 3D video camera, suitable for field work in low- income settings. The images were analyzed using facial analysis algorithms. Normal growth curves were generated for the following facial dimensions: distance between nares and distance from subnasion to upper lip. At birth, 1 year, and 5 years of age the median dimensions were: 13·92, 14·66, and 17.60 mm, respectively for distance between nares, and 10·16, 10.88, and 13·79 mm, respectively for distance from subnasion to upper lip. Modeled facial contours conveniently clustered into three average sizes which could be used as templates for the design of medical instruments.

**Conclusion:**

Capturing of 3D images of infants and young children in LMICs is feasible using portable cameras and computerized analysis. This method and these specific data on Congolese pediatric facial dimensions may assist in the design of appropriately sized medical devices (thermometers, face masks, pulse oximeters, etc.) for this population.

## Introduction

Quantitative facial morphometry has numerous potential medical applications. Examples include measurement of facial dysmorphism for diagnosis of genetic conditions, facial reconstruction in forensic medicine, construction of individualized prostheses in dental and maxillofacial surgery, and design of masks, headgear, respirators, or other medical devices. [1–4] Although numerous studies of facial dimensions have been performed in adults in high-income settings, few data are available for African children.

**Fig 1:**
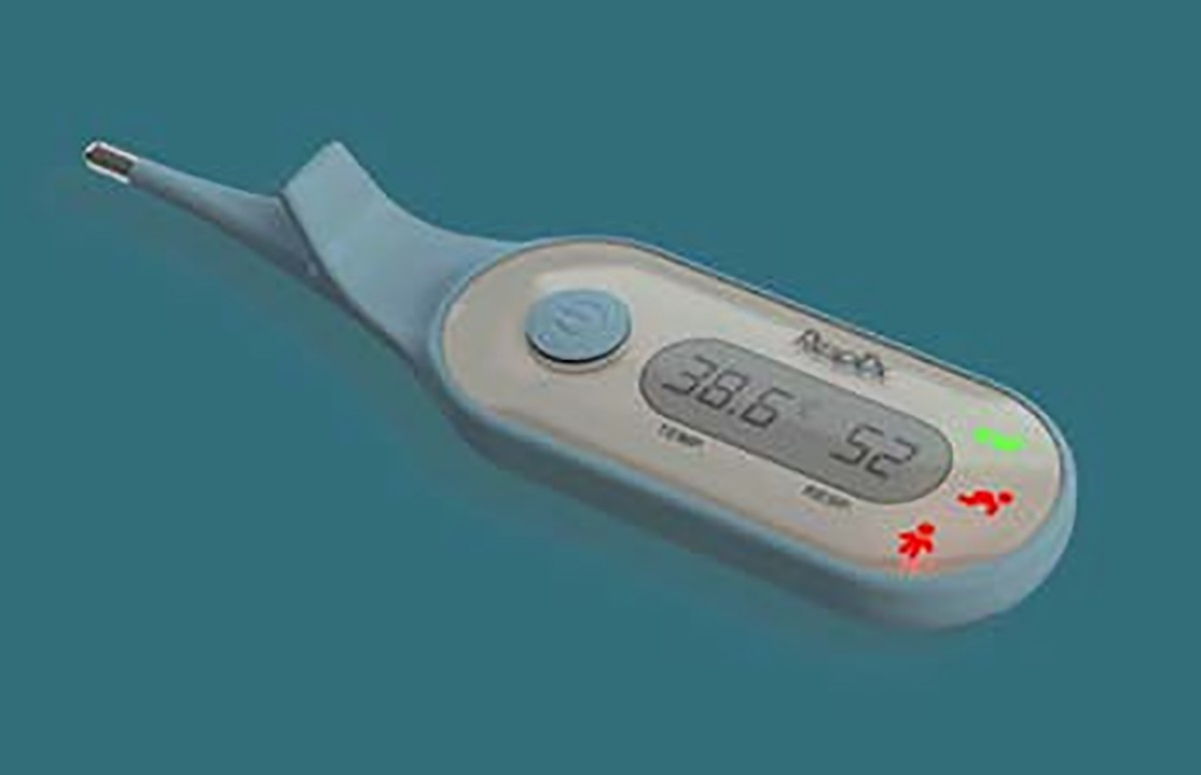
First prototype of the Respimometer.

As one example of a medical device that required quantitative norms for facial dimensions, our own group has previously described a prototype instrument, the Respimometer, for the automated measurement of RR (Fig 1). [5]

The Respimometer was a first prototype oral thermometer-shaped device, equipped with thermistors placed at the nares to measure airflow at the nose and objectively derive the RR from the temperature waveform detected by the thermistors.

In initial testing it become apparent that, while the Respimometer prototype was suitable for Caucasian physiognomies, it may not be optimal for non-Caucasian children living in areas with a high rate of pneumonia mortality such as the Democratic Republic of Congo (DRC). For example, it lacked proper alignment of the temperature sensors to the nose.

Furthermore, as growth and development are associated with rapid changes in facial contours in the very young, more than one size of mouthpiece may be required for children of different ages and sizes. To inform the optimal design of the instrument, anthropometric data from the faces of infants and young children using the device were needed.

Given the importance of the facial structure, the purpose of this study was to obtain facial anthropometric data with respect to ethnic difference and the rapidly changing surface contours and increasing size of faces during the first 5 years of age. Because of the potential application of these findings to design of diagnostic devices for childhood pneumonia, this study was conducted in the Democratic Republic of the Congo (DRC), a country with high pneumonia burden.

## Methods

### Study participants and setting

The study was conducted during the month of June 2017 in Butembo, DRC. The DRC is a low-income country, with a decimated health care infrastructure due to long- standing violent conflict and political instability. The country has one of the highest number of childhood pneumonia deaths worldwide [6]. The population of Butembo is largely Nande, a Bantu ethno-linguistic group. The study was conducted at four health facilities: Centre Hospitalier Universitaire du Graben, Hôpital Général de Matanda, Centre Hospitalier Wanamahika, and Poste de Santé La Guérison. Children in these facilities were either admitted for inpatient care of various conditions or were attending outpatient visits for vaccination or well-child monitoring.

Inclusion criteria included: age 1 month to 5 years, signing a parental consent, and cooperation with the scanning procedure. Exclusion criteria included: any kind of respiratory illness (per parents’ report) in the week preceding enrollment and congenital facial anomalies.

Subjects were recruited on a voluntary and convenience basis. The study was registered at ClinicalTrials.gov (NCT03265756). After obtaining the appropriate Institutional Review Board approval (The Comité d’Ethique du Nord Kivu, and the Research Ethics Board of the University of Alberta) and signed parental informed consent, we obtained 3D scans from infants and young children. All the methods were carried out in accordance with the relevant guidelines and regulations in DRC. The individual in this manuscript has given written informed consent (as outlined in PLOS consent form) to publish these case details.

### Image acquisition

The process started with the capturing of children’s faces using non-contact digitizing structured light technology. Images were acquired using the 3D video camera developed at the Geometric Image Processing (GIP) laboratory at the Technion University in Israel. [7,8] These cameras are portable, accurate, and relatively less costly cameras compared to conventional 3D cameras and were previously used by our group with children in developed countries. [9,10] The cameras use structured light to exploit the deformation of light patterns projected onto an object, and calculate the depth using traditional triangulation. The resulting virtual face model consists of a dense sampling of 3D coordinates (called a point cloud), or triangular meshes representing the children faces. The data was then subjected to clustering, and average virtual face models were created in standard 3D format (CAD or other).

**Fig 2:**
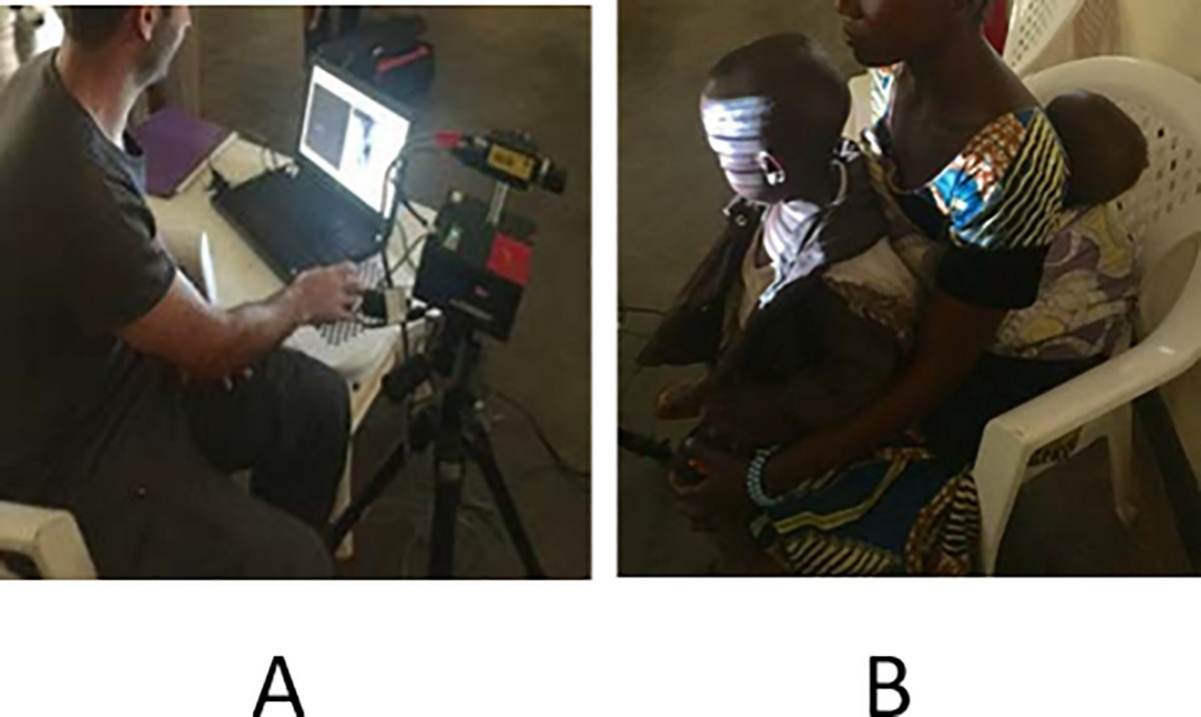
Image acquisition. A. operator, B. scanned child.

Images were obtained in a quiet room with only the operator, caregiver, and child present (Fig 2).

Children were usually seated on a chair or on the lap of the parent or caregiver. Calibration was performed each day before scanning using a standard multi-plane chessboard procedure. The process was semi-automatic whereby the user rotated the chessboard in front of the camera, while the software automatically calculated camera intrinsic and extrinsic parameters. The calibration also provided an estimation of the system's accuracy (~0.2 mm 3D accuracy). All children were scanned at the same distance (70 cm) from the camera, with the same light conditions. Each scan was video-recorded for up to one minute. Images were previewed on site and if necessary scanning was repeated.

All scans were stored on hard drive and were securely transferred to the Technion GIP lab for image analysis.

### Data analysis

The analysis procedure commenced with examining raw videos of 3D facial scans and concluded with clustering of the facial structures into 3 average face masks. Each video clip consists of 100–200 frames. Single video frames were first examined for best frontal orientation. The best frame from each video clip was then oriented into an ideal static position. Coarse pose estimation of head position and orientation was approximated using 4 manually marked (by the operator) landscape points (middle of both eyes, mouth and nose tip).

**Fig 3:**
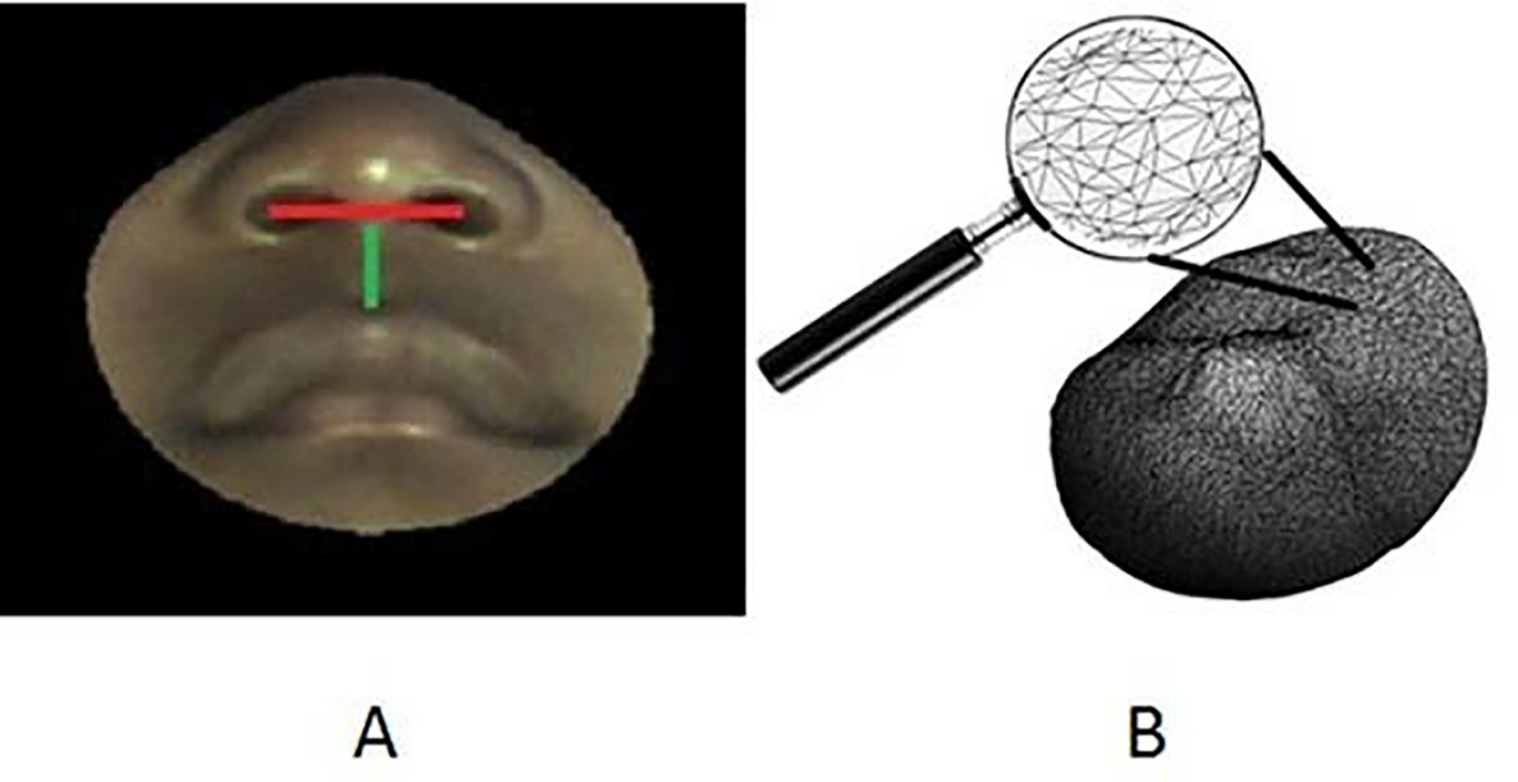
A. distances measured in ROI. B. triangulated face mesh.

For the Respimometer design, the two areas of importance were: 1) The philtrum (upper lip) where a future planned pulse oximeter sensor would be positioned, and 2) The area in proximity to the nares opening where inhalation/exhalation is sensed by the flow sensors. The region of interest (ROI) selected for further analysis encompassed these two areas. The two distances which were measured in these ROIs were the distance between nares and the distance from subnasion to upper lip (Fig 3A).

Each 3D ROI provided several thousand points in 3D space, known as a *point-cloud*. These points, together with the triangles connecting them formed a *triangulated mesh*, or simply *mesh* (Fig 3B).

To align all available scanned meshes we used the iterative closest point (ICP) numerical algorithm. [11,12] This is the standard method for matching geometric structures, estimating the rigid transformation that minimizes the alignment error between the mesh representations of the surfaces. It does so by iteratively calculating the 6 parameters (rotation & translation in 3D) that best aligns a source mesh to a target mesh. The ICP method is capable of aligning unordered sets of points, with different sizes, and handles occlusions and missing data. It starts with an initial estimate with regard to the orientation and position of one surface with respect to the other. In this case, the initial guess is derived from the rough facial orientation of the face models, extracted from the location of eyes, nose and mouth. ICP Accuracy is determined by averaging the distances of each point on the transformed source mesh to the closest point on the target mesh.

NRICP (Non Rigid ICP) is an extension of the basic ICP, allowing each vertex to move independently (having its own rotation and translation parameters), as previously suggested. [13,14] Contrary to the basic ICP, where the meshes retain their fixed structure, the non-rigid version allows deformation of the surfaces. Usually, NRICP requires a set of feature points as anchors. In this case, feature points were hard to obtain in many faces, especially in the relevant part of the face (containing upper lip and nose). Therefore, an NRICP version without landmarks was used. An important benefit of the NRICP is that an ordered constant sized point-cloud (or mesh) with ordered vertex indices is deformed from source to target, such that after applying the transformation we obtained the same vertex ordering on the target mesh, defining a one-to one correspondence from each point/triangle on the source to their counterpart on the target mesh.

Once all face meshes were aligned by the rigid ICP, an average template mesh was constructed, and deformed by the NRICP into each of the face models. In practice, the 'average template' was not available a-priori. Instead, one of the faces was selected as anchor substituting for the template. After the first iteration, aligned fixed sized and ordered meshes were obtained, and an average facial mesh was created and used as a template for the next iteration.

Measurement of shape similarity between two facial models was defined by the mean Euclidean distances between all ordered points of the models after NRICP alignment. Variance among all face models was formalized by a NxN difference matrix, N being the number of facial models. This difference matrix is an algebraic representation of the measured alignment of each facial model to all the others, its entries being pairwise alignment measurements.

The K-means algorithm [15] was then applied to the difference matrix to cluster the facial models into three groups, and the average model for each group was created, also represented as a triangular mesh. The three average facial templates are such that for the whole population, the sum of alignment errors from each model to its closest average template is minimized.

### Role of the funding sources

The funders had no role in study design, data collection, data analysis, data interpretation, writing of the report, or decision to submit the article for publication.

## Results

321 children participated in the study and 3D images were obtained. Of these, 287 images were selected for further processing. Reasons for exclusion of the remaining images were: noisy image, moving, crying, or covered child, failure to meet strict numerical convergence requirements. The median age of these 287 children was 3·5 years (range 1 month to 5·5 years) and 51% were female.

**Fig 4:**
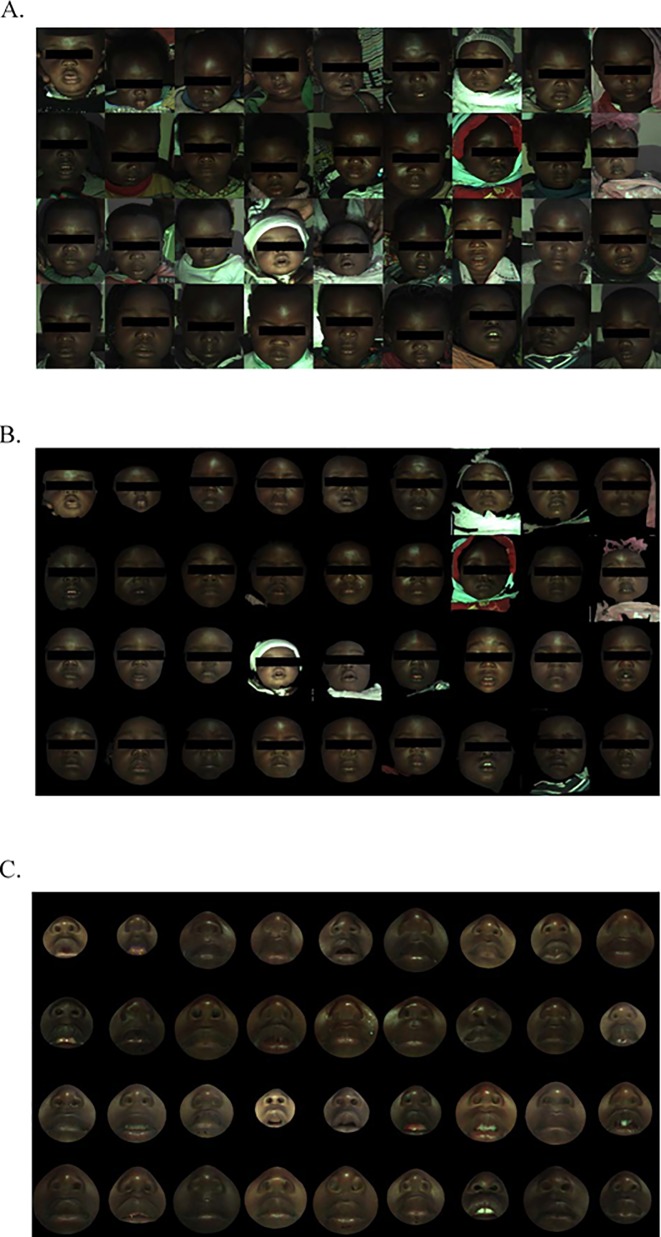
Selected examples of 36 Congolese children with 3D facial photographs. A. Raw image. B. Processed image, bringing face into forward alignment. C. Region of interest (ROI), restricted to nose and mouth, appropriate for measurement of distance between nares and distance from subnasion to upper lip.

A sample of 36 representative facial images is shown in Fig 4 in their original form (Fig 4A), after applying the alignment algorithm (Fig 4B) and after cropping the ROI (Fig 4C, mouth and nose).

Alignment accuracy was measured by averaging distances of each point on a source facial model to the closest point on a single template model. The mean accuracy for all faces was 1·04 mm (SD = 0·43) for the selected ROI.

The mean (SD) distance from the subnasion to upper lip was 10·04mm (1·60), 11·88mm (1·89), and 13·86 (1·81) in the small, medium, and large clusters, respectively. The mean (SD) distance between the nares was 13·44 mm (1·50), 15·90 mm (1·56), and 17·55 (1·79) in the small, medium, and large clusters, respectively.

**Fig 5:**
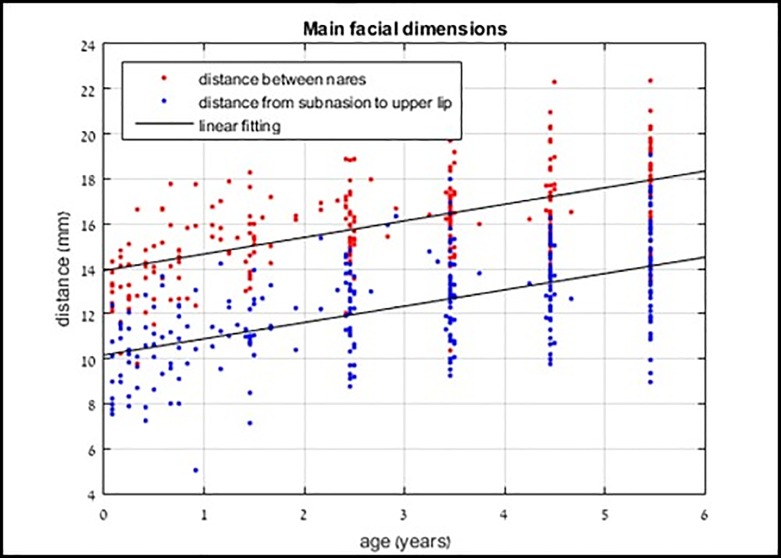
Main facial dimensions by age with linear approximation for each of the two measured distances.

Fig 5 presents these measurements, by child age. The correlation coefficient (Spearman's rho) of the linear approximations between age and the distance between the nares was ρ = 0·60, and ρ = 0·56 between age and the subnasion-upper lip distance. The associated *p*-values for both linear approximations were all lower than 0·001, indicating a strong correlation between child age and each of these distances.

**Fig 6:**
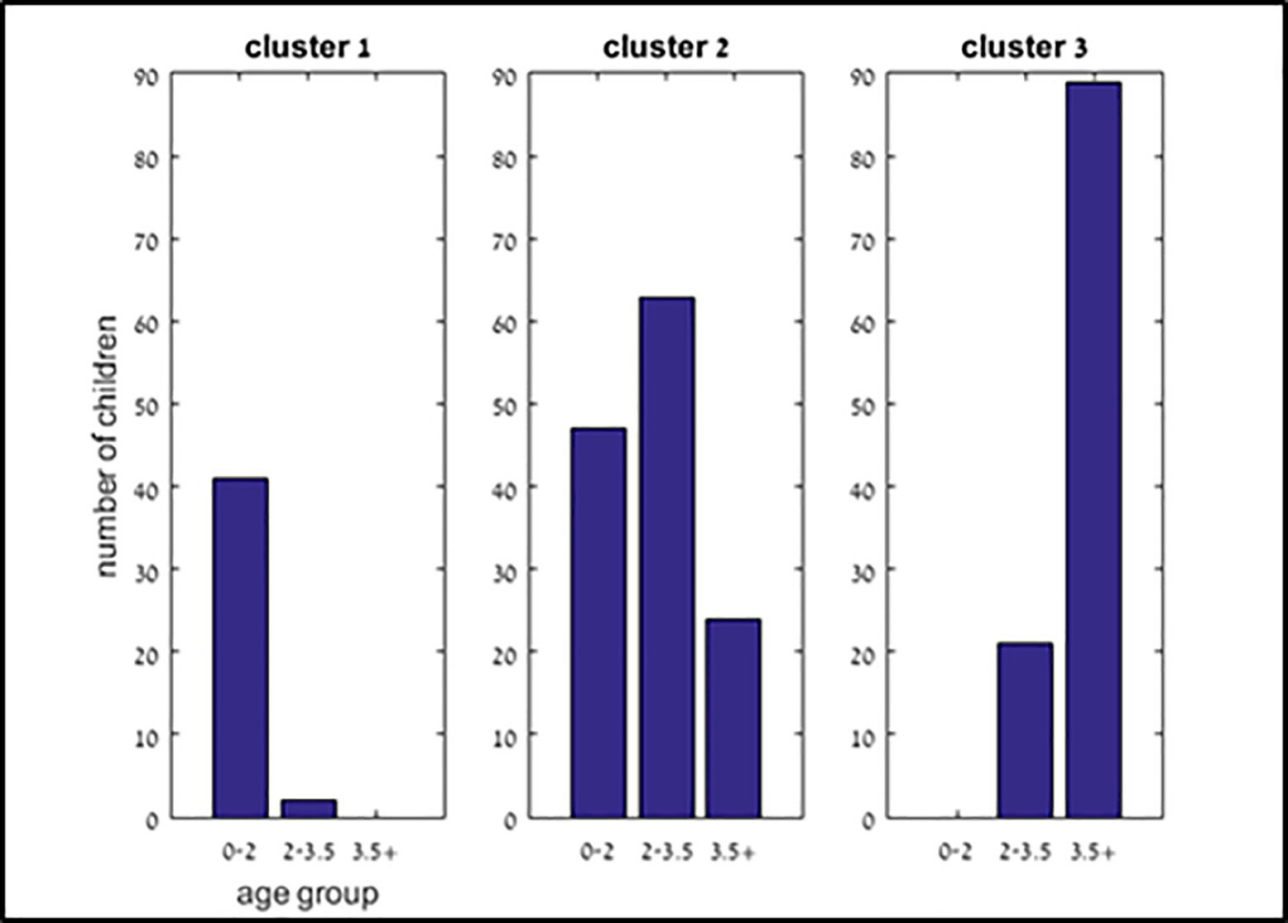
Age distribution in each cluster.

The virtual facial meshes were grouped into three clusters, by three different criteria:

(1) according to shape similarity (as defined by the measurement of alignment between each model to all the others) (2) by pre-defined age groups (0–2 years, 2–3·5 years, over 3·5 years), and (3) by the two previously mentioned distances. Fig 6 depicts the clustering by shape similarity, and shows the age distribution of children within each cluster.

**Fig 7:**
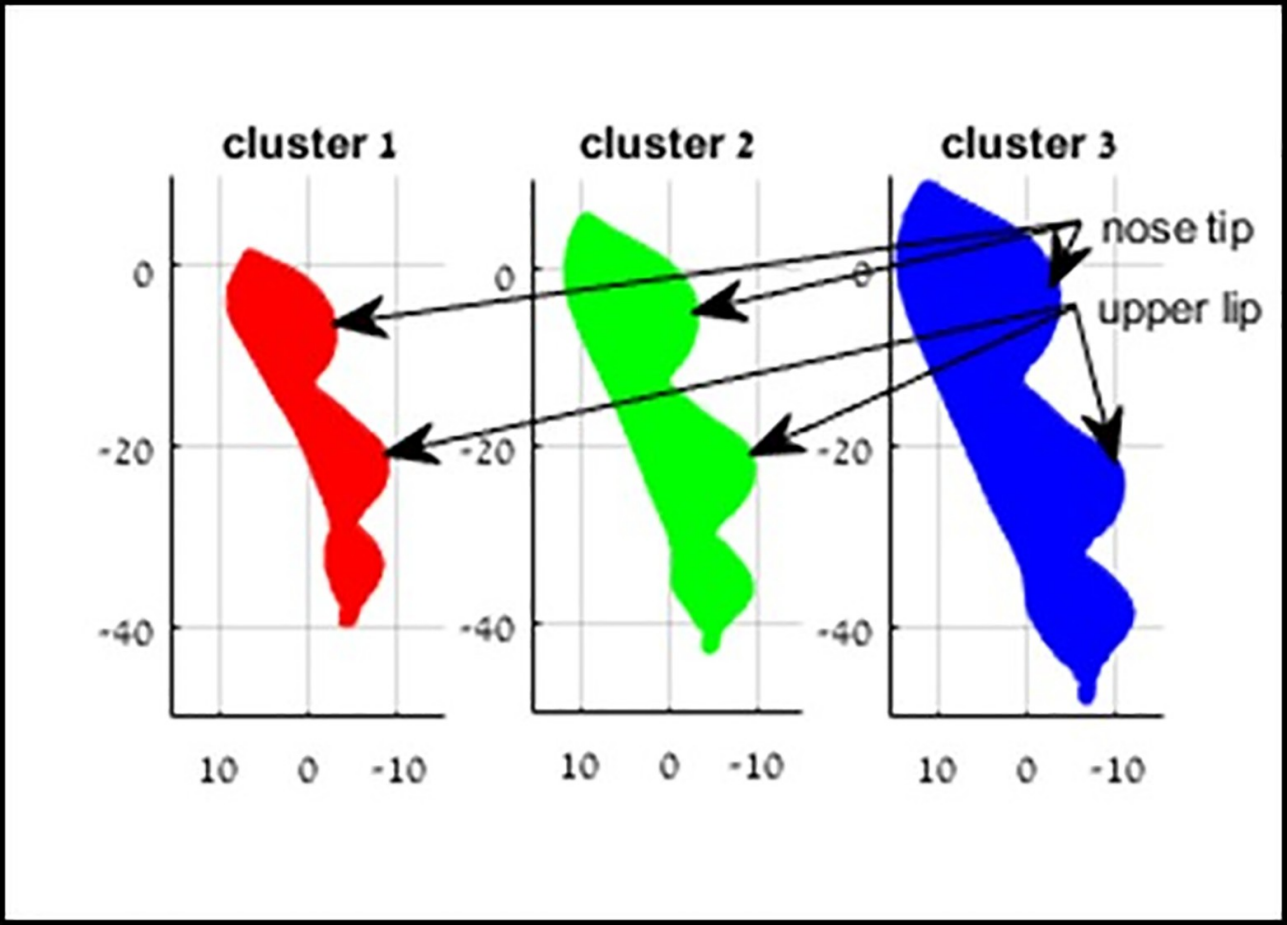
Average masks of 3 clusters. Left–small size, right–larger size. Axis units are in m”m.

Average facial contours over the regions of interest for each of the clusters (by the shape similarity alignment) are displayed in Fig 7. As expected, more younger children were included in the cluster of smaller faces and more older children were included in the cluster of larger faces. (*p* = 0·012, 0·001, 0·035 for the small, medium and large sized average models, respectively).

It should be noted that when clustering by different criteria, the same aligned face models were used, and the alignment accuracy was calculated comparing *each individual* face model to the average model of the cluster it belongs to.

When comparing clustering by shape similarity, age groups, and by two main distances, we obtained the following mean (SD) alignment errors: 1·68mm (0·85), 1·84mm (0·95) 1·96mm (1·04), and respectively. This implies that clustering by shape similarity results in the least alignment error.

### Application of facial morphometric data to the design of a medical device

**Fig 8:**
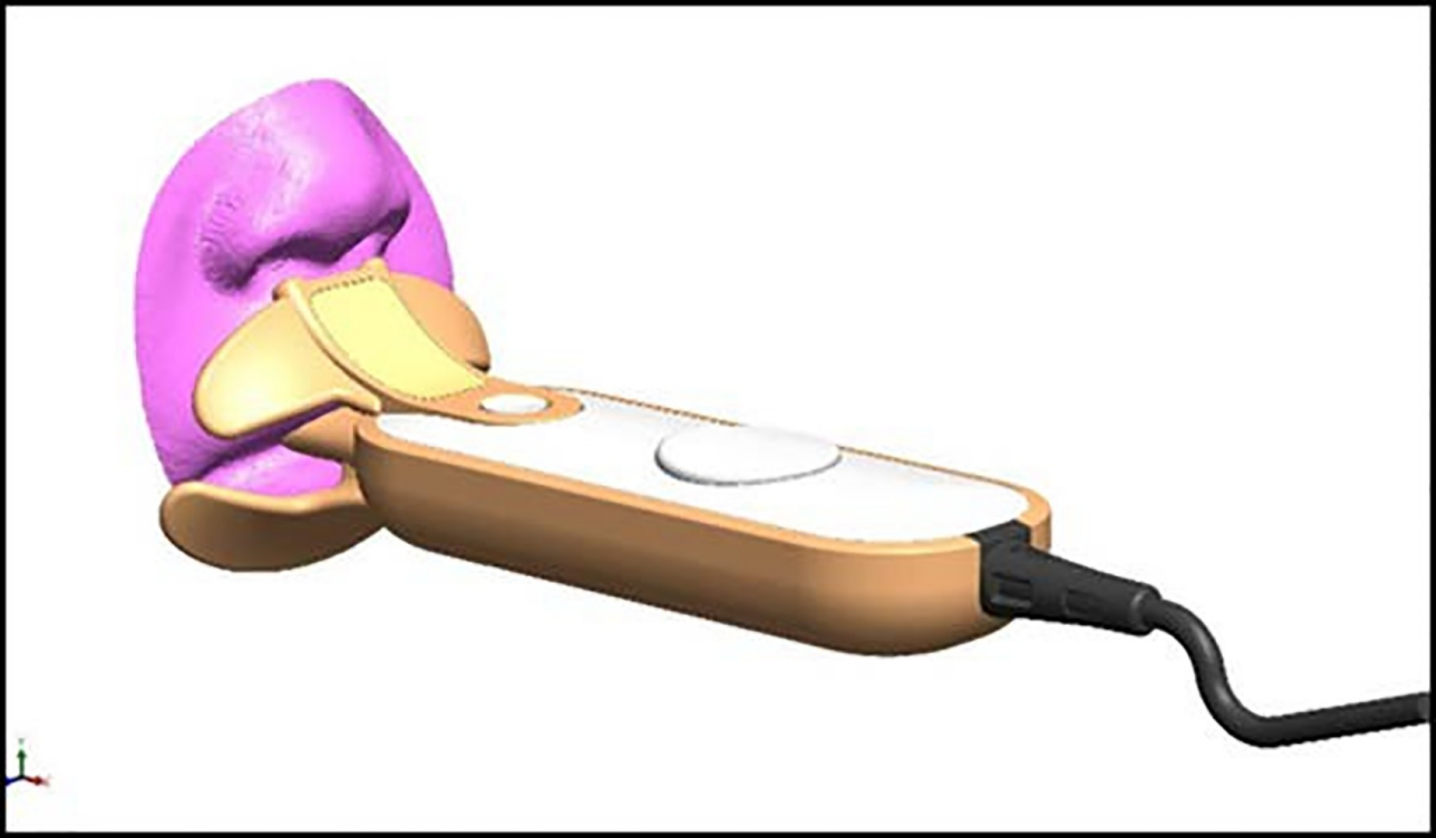
Newly designed device for measuring temperature, respiratory rate and oxygen saturation.

We next applied the average facial contours to improve the design of our prototype instrument for the automated measurement of RR, the Respimometer [5] The distance from the subnasion to upper lip and the distance between the nares were used as key design parameters for the length and width of the upper lip flap of the device, designed to position the thermistors at the nasal outlet for optimal detection of temperature waveform (Fig 8).

## Discussion

In designing medical devices that require a fit to oral or facial structures, it may be important to consider variation in morphometric properties that occur with ethnicity and/or age. The potential for *ethnic* difference in facial anthropometry in Africa was recently suggested. [16,17]. In the present study we acquired 3D images of 287 ethnically Nande children age 1–5 years in the DRC. The images obtained from the children were utilized in ~90% of the cases, producing accurate facial models, aligning and registering them, grouping them into several clusters, and creating average virtual facial masks for each group. Such average virtual face models may be useful for design of medical devices such as the Respimometer. [5] These virtual face models are now being further converted to stereo-lithograpy (STL) by which computerized 3D models are to be constructed. These models serve as the basis for instrument design.

The approach described in this paper incorporating a portable 3D camera with pre- programmed analysis, can be used to analyze the shape of facial contours of various ethnic groups, and thereby enable the improved design of various medical devices, such as face masks. In this way, designs can be tested and validated against a range of facial shapes so as to ensure a good fit universally across a diverse set of populations.

There are few previous studies of facial dimensions in young children in Africa. A recent study used a hand operated sliding caliper to measure ear lengths, ear projection and face height in Zimbabwean children. [18] Cephalometric radiographs of children in Africa have been commonly used for various facial measures for example in planning future orthodontic and orthognathic surgery. [19,20] The main disadvantage of this methodology is the use of radiation. Newer studies have used hand-held laser scanners to obtain three-dimensional facial coordinates in Sudanese children and in Tanzanian population. [16,17] The cost of these scanners is a disadvantage. Furthermore, these technologies measure a small set of Euclidean distances among landmark points, and not a geometric estimation of surface to surface alignment. When designing a device that should fit the surface, Euclidean distances among anchor points is important, but more so is the intrinsic structure of the surface, as well as a metric minimizing deformation between surfaces.

The current technology described in the present study offers an alternative non- radiation low-cost method to obtain these important facial dimensions. The portability of the equipment, its cost, and the capability to accurately capture facial structure are additional important advantages. While the GIP 3D camera we have used in this study has been used in developed countries. [9,10] this is the first description of its use in a LMIC setting. In remote locations the hosting laptop could be charged by a solar charger, while the camera can be powered by the laptop or handheld device. This would make the system completely independent from a reliable electric power grid.

Our study is subject to several limitations. Several problems were identified during the image processing–noisy data, low reflectance of dark colored subjects, incomplete region of interest in the image (for example–nostrils missing in image and interpolated), field of view (FOV) larger than actually needed (ROI should be area surrounding nose + mouth, not whole face) and unsuitability of automatic landmark detection. NRICP without landmarks has proved possible, although with some minor limitations (non-convergence in a few cases).

In summary, normal values of the facial dimensions of Congolese children provided here may assist in the accurate and age-appropriate design of pediatric medical equipment. Furthermore, we have described a generalizable method for obtaining facial anthropometry data in a field setting with portable equipment and no radiation.
